# Predicting Nipple Necrosis with a “Lights-on” Indocyanine Green Imaging System: A Report of Two Patients

**DOI:** 10.1055/s-0043-1777068

**Published:** 2024-04-04

**Authors:** Ellen C. Shaffrey, Steven P. Moura, Sydney Jupitz, Trevor Seets, Tisha Kawahara, Adam Uselmann, Christie Lin, Samuel O. Poore

**Affiliations:** 1 Division of Plastic Surgery, University of Wisconsin School of Medicine and Public Health, Madison, Wisconsin; 2 OnLume Surgical, Madison, Wisconsin

**Keywords:** fluorescence imaging, intraoperative, ambient light compatible, breast reconstruction, nipple necrosis

## Abstract

Nipple–areolar complex (NAC) necrosis is a devastating complication in nipple-sparing mastectomies (NSMs) that significantly impacts patient's quality of life. The use of fluorescence angiography for intraoperative assessment of mastectomy skin flap perfusion in NSM has been successfully described and can be utilized to help guide surgical decision-making. Recently, a novel fluorescence-guided surgical imager was developed, OnLume Avata System (OnLume Surgical, Madison, WI), which provides intraoperative evaluation of vascular perfusion in ambient light. In this case report, we describe the use of OnLume fluorescence-guided surgery technology to help aid in clinical decision-making for two breast reconstruction cases with concern for intraoperative nipple hypoperfusion.

## Introduction

Nipple necrosis unfortunately complicates 3 to 37% of nipple-sparing mastectomies (NSMs) making preoperative discussion critical as postoperative development of this complication can result in emotional stress, prolonged surgical management, and increased cost. 1 Known surgical risk factors for nipple–areolar complex (NAC) necrosis include higher mastectomy weight, thinner mastectomy skin flaps, and a periareolar incision. 2 3 However, compared to women who undergo skin-sparing mastectomies, NSMs provide significant psychological benefits such as improved postoperative body image and breast satisfaction. 4

Along with clinical judgment, intraoperative assessment of nipple perfusion during NSMs using fluorescence-guided surgery (FGS) indocyanine green (ICG) angiography can be performed, with prior studies demonstrating a strong correlation between ICG findings to areas of the mastectomy skin that ultimately develop skin necrosis. 1 5 Implementation of ICG angiography in breast cancer surgery has also resulted in a decrease in mastectomy skin necrosis rates, with Duggal et al demonstrating a reduction in necrosis requiring reoperations from 14.1 to 5.9% ( *p* = 0.009). 6 However, some currently available ICG systems, when operated in the presence of ambient light, experience light contamination, thereby compromising image quality and detection sensitivity of the near-infrared signal from the dye. 7 Notably, the U.S. Food and Drug Administration recently cleared the OnLume Avata™ System (OnLume Surgical, Madison, WI). The FGS technology of the Avata System utilizes transient lighting to allow for simultaneous capture and display of color and monochrome fluorescence images in ambient light without impact on image quality. This allows for high-definition resolution, real-time overlay visualization and can be utilized at a variety of working distances thanks to its electronic zoom and focus features. The Avata System's large range of field of views, variable working distance, and adjustable camera angle allows it to easily adapt to different regions of interest and integrate into a variety of surgical scenarios. In this case report, we describe the use of the OnLume Avata System to predict nipple necrosis and help guide surgical and postoperative decision-making in two patients undergoing bilateral breast reconstruction with NSM.

## Cases

### Patient 1

The first patient was a 48-year-old, nonsmoking woman with ductal carcinoma in situ in bilateral breasts. On preoperative exam, she was noted to have a body mass index (BMI) of 29.6, excess subcutaneous abdominal tissue, and size B cup breasts with minimal ptosis. Given her cancer characteristics and physical exam findings, we determined that she was a candidate for NSMs. The patient underwent bilateral NSM with immediate breast reconstruction using autologous deep-inferior epigastric artery perforator (DIEP) flaps performed by the senior author (S.O.P.).

**Fig. 1:**
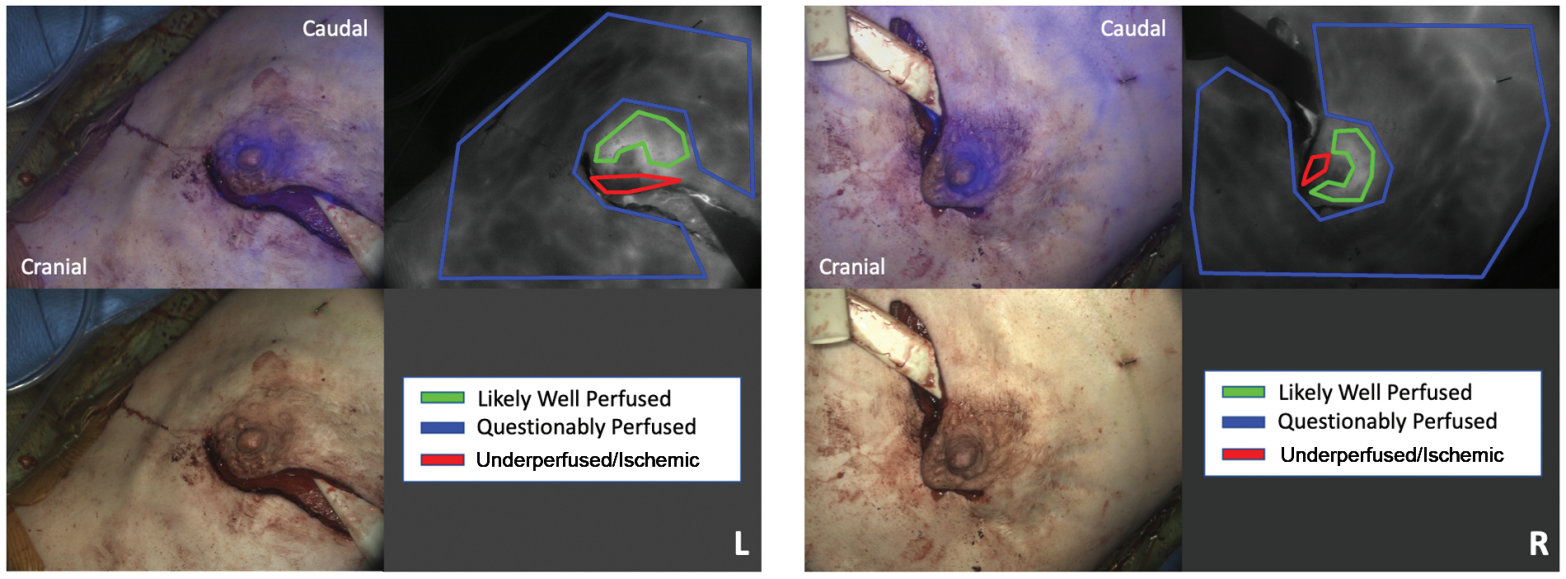
Patient 1 bilateral mastectomy skin flap fluorescence (top left: blue false-color fluorescence on color image, top right: monochrome) and clinical color (bottom left) images captured intraoperatively demonstrating focal areas of ischemia to the nipple–areolar complex with global hypoperfusion of her remaining mastectomy skin flaps. Post hoc surgeon annotations: green, “likely well perfused;” blue, “questionably perfused;” red, “underperfused/ischemic.”

Both NSMs were performed with a periareolar incision. Her mastectomy weights were 552 and 534 g from the right and left breast, respectively. Before the DIEP flap inset, there was a clinical concern for hypoperfusion of the nipples bilaterally. Therefore, vascular perfusion imaged in real time using the OnLume Avata System, which demonstrated global hypoperfusion of her mastectomy skin flaps and dark, poorly perfused areas of the NACs ( Fig. 1 ). Given these findings, the senior author elected to bank a skin paddle on the DIEP flap below the NACs. For the first two postoperative days, to help improve vascular perfusion, nitropaste was applied to the bilateral mastectomy skin flaps and the NACs. Despite this intervention, she continued to have progressive worsening of NAC ischemia in both nipples, ultimately developing full-thickness necrosis of the entire NAC in both breasts by postoperative day 3. Prior to discharge, the senior author (S.O.P.) reviewed the intraoperative ICG findings, confirmed the irreversible necrosis of the NAC, and discussed the need for surgery within the coming weeks. Approximately 3 weeks after her initial surgery, the patient underwent surgical excision of necrotic nipples and inset of banked DIEP skin flap. Five months later, the patient had nipple reconstruction for both breasts to complete her reconstructive course.

### Patient 2

The second patient was a 40-year-old, nonsmoking woman who presented for a discussion regarding breast reconstruction options with right breast lobular carcinoma in situ. The cancer was identified within her breast tissue specimens after undergoing a breast reduction 3 months earlier. On preoperative exam, she was noted to have a BMI of 34.0, excess subcutaneous abdominal tissue, and size C cup breasts. The patient strongly preferred to attempt NSMs despite her larger breast size. After a thorough discussion regarding the risks and benefits, both she and her surgeons agreed to attempt NSMs with immediate DIEP flaps, given that her prior breast reduction could result in a delay phenomenon for her skin. The same reconstructive team performed the DIEP flaps.

**Fig. 2:**
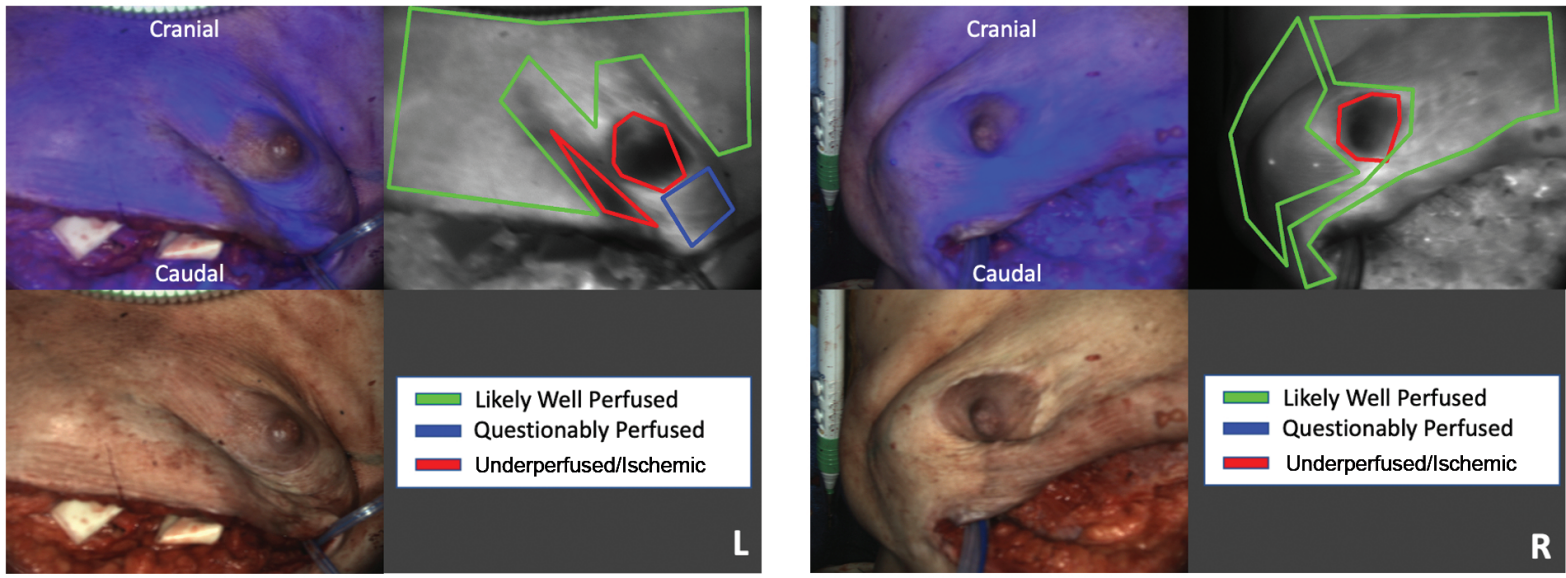
Patient 2 bilateral mastectomy skin flap fluorescence (top left: blue false-color fluorescence on color image, top right: monochrome) images captured intraoperatively demonstrating ischemia to the nipple–areolar complex bilaterally but robust perfusion of the remaining mastectomy skin flaps. Post hoc surgeon annotations: green, “likely well perfused;” blue, “questionably perfused;” red: “underperfused/ischemic.”

The NSMs were performed with an inframammary incision bilaterally. Her mastectomy weights were 836 and 808 g. There was clinical concern for bilateral NAC ischemia; therefore, an assessment of vascular perfusion was performed utilizing the OnLume Avata System. Her imaging findings showed bilateral hypoperfusion of the NAC and mastectomy skin immediately below the NAC. However, the remainder of the mastectomy skin flaps were well-perfused ( Fig. 2 ). To preserve the NAC on both breasts, the senior author elected to remove the lower, hypoperfused mastectomy skin and inset the DIEP flap with a skin paddle that could be used for nipple reconstruction. Postoperatively, nitropaste was ordered for the bilateral NACs and continued until postoperative day 3. The patient had progressive resolution of hypoperfusion with normal capillary refill of both areolas noted on postoperative day 2. On the discharge date, necrosis was localized to the tips of both nipples, and no additional surgical interventions for the nipples were required.

### Comparison of OnLume Findings

**Fig. 3:**
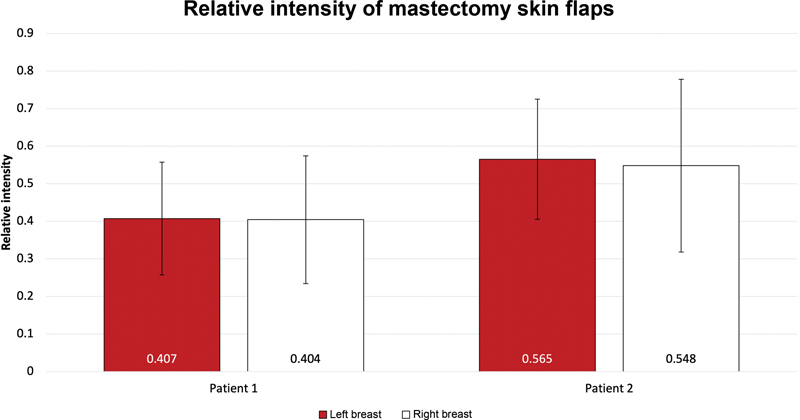
Comparison of relative fluorescence intensity values between patients. Patient 1 ultimately developed full-thickness nipple–areolar complex necrosis requiring additional surgical intervention, while Patient 2 had recovery of perfusion.

Institutional Review Board (IRB) approval and subject consent was obtained prior to patient data collection (ID: 2020-0906). Informed consent was obtained from both patients to utilize clinical photos and information for this manuscript. Post hoc analysis of OnLume Avata images for both patients, obtained at the point of maximal perfusion, was performed by a plastic surgeon not involved in the patient's initial operation (E.C.S.) to allow for unbiased analysis of the images. This surgeon had significant prior clinical experience with reviewing fluorescence images for vascular perfusion. Areas of the mastectomy skin and NAC were subjectively assessed and outlined as either “likely well perfused,” “questionably perfused,” or “underperfused/ischemic.” Relative fluorescence intensity values were normalized between images to allow for comparison and correlate to the degree of vascular perfusion within the tissues. *Patient 1* , who ultimately developed full-thickness NAC necrosis in both breasts, demonstrated an overall lower mean relative intensity of their mastectomy skin flaps (left breast: 0.41, right breast: 0.40), compared to *Patient 2* , whose nipples ultimately survived (left breast: 0.57, right breast: 0.55; Fig. 3 ). Furthermore, the majority of *Patient 1's* mastectomy skin flap was identified to be either “questionably perfused” or “underperfused/ischemic” despite portions of her NAC being labeled as “likely well perfused” ( Table 1 ).

**Table 1:** Relative areas of vascular perfusion

		NAC necrosis?	Relative area (%)		
			Likely well perfused	Questionably perfused	Underperfused/Ischemic
Patient 1	Left breast	Yes	5.4	92.8	1.8
	Right breast	Yes	3.1	96.3	0.6
Patient 2	Left breast	No	83.6	5.6	10.8
	Right breast	No	92.5	0.0	7.5

Abbreviation: NAC, nipple–areolar complex.

## Discussion

Nipple loss in patients undergoing NSM is an unfortunate complication, given that it is devastating to the patient and decreases their quality of life after reconstruction. Methods to predict NAC necrosis are critical to help guide intraoperative decision-making and postoperative care. In this report, we describe the use of a high-resolution, ambient light-compatible FGS technology to evaluate two patients with intraoperative nipple hypoperfusion and demonstrate differences in their mastectomy flap vascularity. Interpretation of these images and close postoperative monitoring permitted informed patient counseling when deciding if additional surgical management was required.

When reviewing the OnLume Avata fluorescence images of both patients' mastectomy skin flaps, there is a clear difference in the relative intensities and areas of adequate perfusion. Despite *Patient 2's* NAC appearing to have a greater area of hypoperfusion, the majority of her mastectomy skin flaps were well-perfused, which may have contributed to the nipples' ultimate survival. Furthermore, the senior author made the intraoperative decision to remove the small area of hypoperfused mastectomy skin, just inferior to the nipple, with inset of the DIEP flap with a skin paddle. This is in contrast to *Patient 1* , who not only had areas of ischemia identified on the NAC but was also noted to have hypoperfused mastectomy skin flaps. The senior author ultimately made the intraoperative decision to bank a large area of DIEP skin in order to perform nipple reconstruction at a later date. Sadly, this was eventually needed, prolonging the patient's reconstructive course.

ICG imaging, combined with clinical assessment, results in high sensitivity in predicting areas of nipple necrosis. 1 Its routine use has been shown to decrease the incidence of complications related to mastectomy skin necrosis, as described by Duggal et al. 6 However, unlike traditional FGS technology, the OnLume Avata System allows for the simultaneous assessment of the clinical anatomy and vascularity, given that both fluorescence and color images can be displayed at video rate in real time with the operating room lights on, therefore potentially increasing its sensitivity to identify areas of underperfusion. Furthermore, the Avata System provides a repeatable and reproducible fluorescence quantitation, as described by Seets et al, whereas the current SPY-Elite and SPY-PHI systems cannot. 8 9 Additionally, there are potential cost savings with implementation of the OnLume Avata System. Despite their limitations, use of previous generation fluorescence systems to assess vascular perfusion in autologous breast reconstruction operations has resulted in a decreased incidence of revision operations, ultimately saving patients an average of $1,400 to $9,550. 5 10 Given that the OnLume System provides even greater reliability, the potential cost savings could be theoretically larger.

Lastly, individual patient characteristics and surgical techniques can influence the incidence of nipple necrosis in patients undergoing NSMs. Patient characteristics such as smoking history, prior radiation, increasing BMI, and large ptotic breasts have demonstrated an increased risk. 11 12 Given that radiotherapy is recommended in more advanced cancers or those with high-grade features, that may also result in a greater risk of nipple necrosis. However, this was not the case for either of our patients who were diagnosed with low-grade in situ cancers. Regarding surgical risks, an extensive systematic review by Lee and Mun demonstrated that incisions involving the NAC, specifically periareolar incisions and mastectomies performed with diathermy, resulted in higher rates of nipple loss. 13 Additional studies have also noted greater mastectomy specimen weight and thinner mastectomy skin flaps also increase the risk of nipple necrosis. 14 15 Regarding our patients, despite *Patient 2* having larger breasts, a higher BMI, and greater mastectomy tissue resection weight, the use of an inframammary incision may have been a protective factor in her NAC ultimately recovering its perfusion. Furthermore, her history of prior breast reduction also may have resulted in the delay phenomenon with enhanced vascularity to the skin flaps.

We recognize that tissue perfusion is dynamic, and therefore, a static measurement may not be entirely predictive of necrosis. Therefore, as with previous fluorescence technologies, FGS imaging should be used as a supplement to clinical assessment. Unlike conventional “lights-off” intraoperative angiography systems, the OnLume Avata imaging system was easily integrated into the surgical workflow to permit the detection of nipple ischemia and assisted with real-time intraoperative decisions in the setting of ambient light.
